# Anesthetic management with remimazolam in very elderly patients undergoing hybrid surgery of transcatheter aortic valve implantation plus off-pump coronary artery bypass grafting: report of two cases

**DOI:** 10.1186/s40981-023-00662-4

**Published:** 2023-10-25

**Authors:** Takafumi Oyoshi, Yuki Mitsuta, Yumiko Uemura, Koichiro Tajima, Naoyuki Hirata

**Affiliations:** 1 Department of Anesthesiology, Kumamoto University, Graduate School of Medical Sciences, Kumamoto, Japan; 2 Department of Anesthesiology, Japanese Red Cross Kumamoto Hospital, Kumamoto, Japan; 3 Department of Anesthesiology, Minamata City General Hospital and Medical Center, Kumamoto, Japan

**Keywords:** Remimazolam, Aortic stenosis, Elderly, Hemodynamics, Hybrid surgery, Transcatheter aortic valve implantation, Off-pump coronary artery bypass grafting

## Abstract

**Background:**

Remimazolam is a short-acting benzodiazepine with small circulatory depression. We used remimazolam for general anesthesia management in two very elderly patients undergoing hybrid surgery of transcatheter aortic valve implantation (TAVI) plus off-pump coronary artery bypass grafting (OPCABG).

**Case presentation:**

A 96-year-old man (case 1) and a 92-year-old woman (case 2) had complex coronary artery disease (CAD) and severe aortic stenosis (AS) and were scheduled for TAVI plus OPCAB. Anesthesia in both patients was induced with 6 mg/kg/h remimazolam and fentanyl and maintained with 0.3 mg/kg/h and 0.5 mg/kg/h remimazolam and fentanyl, respectively. Although catecholamines were required, we successfully maintained circulation during the induction of anesthesia and the procedures of OPCAB and TAVI. Both patients were discharged without complications.

**Conclusion:**

Remimazolam can be a useful option for safe general anesthesia in very elderly patients when performing hybrid surgery.

## Background

The number of elderly patients with both aortic stenosis (AS) and coronary artery disease (CAD) is increasing with the aging of the population. It is estimated that CAD is present in up to 75% of patients undergoing surgical aortic valve replacement or transcatheter aortic valve implantation (TAVI) [1]. Hybrid surgery of TAVI plus coronary artery bypass grafting (OPCAB) is a valid option in these AS patients with high surgical risk and complex CAD as the adverse effects of cardiopulmonary bypass can be avoided [2–4].

Patients with AS coexisting with CAD have increased oxygen demand resulting from myocardial hypertrophy, while oxygen supply is deficient because of cardiac output restriction and coronary artery stenosis. General anesthetics could cause hypotension due to cardiac depression and vasodilation and subsequent fatal myocardial ischemia. Furthermore, intraoperative hypotension can be caused by manipulations of TAVI and OPCAB. Therefore, anesthesia management for hybrid surgery is very challenging.

Remimazolam is a short-acting benzodiazepine with less hemodynamic effect than inhalational anesthetics and propofol [5, 6]. Remimazolam has also been reported to be safe for use in high-risk elderly patients [7]. We report two cases of general anesthesia management with remimazolam in very elderly patients undergoing TAVI plus OPCAB hybrid surgery.

## Case presentation

Written informed consent was obtained from each patient for the publication of this case report.

### Case 1

The patient was a 96-year-old man (height, 160 cm; weight, 47.4 kg) with an enlarged abdominal aortic aneurysm of 53 mm in size, for which invasive treatment was considered. In a preoperative medical examination, transthoracic echocardiography showed severe AS with an aortic valve peak velocity of 4.2 m/s, calculated aortic valve area of 0.52 cm^2^ and circumferential left ventricular wall thickening. Left ventricular ejection fraction was maintained at 70.4%. Coronary angiography revealed a complex three-vessel coronary artery lesion with a SYNTAX score of 34. In addition, he had anemia with a hemoglobin level of 8.2 g/dL, hypertension, dyslipidemia, and hypokalemia with a potassium level of 2.9 mEq/L. After thorough consultation with the Heart Team, hybrid surgery of TAVI plus OPCABG was scheduled in preference to abdominal aortic aneurysm surgery. The surgical strategy was to first perform revascularization of the left anterior descending artery (LAD) with left internal thoracic artery bypass followed by TAVI via the trans-aortic approach and then perform complete revascularization by bypassing the right coronary artery and left circumflex artery.

**Fig. 1:**
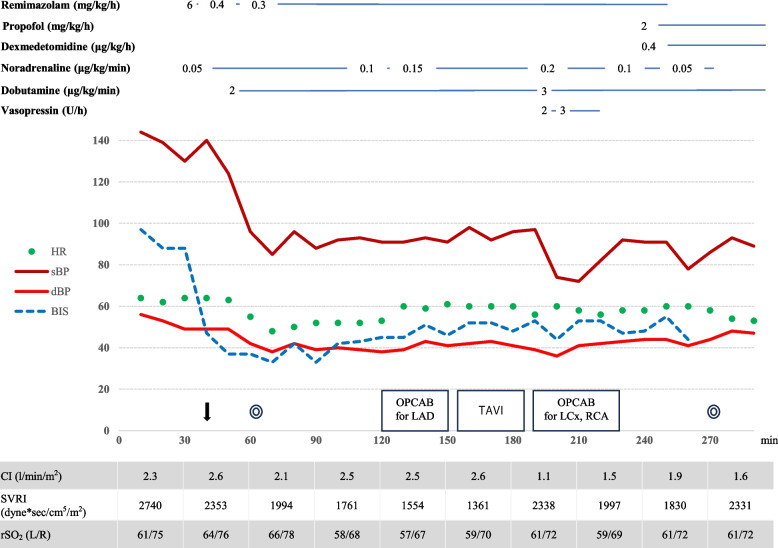
Anesthesia record for case 1. The down arrow represents intubation. Double circles represent the start/end of surgery. HR: heart rate; sBP: systolic blood pressure; dBP: diastolic blood pressure; BIS: bispectral index; CI: cardiac index; SVRI: systemic vascular resistance index; rSO_2_: regional cerebral oxygenation measured by near-infrared spectroscopy; CABG: coronary artery bypass grafting; TAVI: transcatheter aortic valve implantation; LAD:left anterior descending; LCx: left circumflex artery; RCA:right coronary artery

An oximetry catheter (PreSep; Edwards Life Sciences, LLC, Irvine, CA, USA) and a continuous arterial pressure line with hemodynamic monitoring (FloTrac; Edwards Life Sciences, LLC, Irvine, CA, USA) were inserted while the patient was awake. A BIS device and a near-infra-red spectroscopy device (INVOS 5100; Somanetics, Troy, MI, USA) were fitted in addition to standard monitoring. General anesthesia was induced with a total of 200 µg of fentanyl in divided doses and remimazolam at 6 mg/kg/h along with continuous administration of noradrenaline at 0.05 µg/kg/min. After the patient lost consciousness, 0.6 mg/kg rocuronium was administered. No significant circulatory changes were observed during tracheal intubation. Anesthesia was maintained with a total of 1000 μg fentanyl in divided doses and remimazolam was adjusted to 0.3–0.4 mg/kg/h to achieve BIS values between 40 and 60. Hemodynamic change during surgery is shown in Fig. 1. During coronary artery bypass to the LAD, hemodynamics were stable with CI 2.6 L/min/m^2^ and SVRI around 1300 dyne sec/cm^5^ with the administration of 0.15 μg/kg/min noradrenaline and 2 μg/kg/min dobutamine. TAVI using a balloon-expandable bioprosthetic valve (sapien3; Edwards Lifesciences, Irvine, CA, USA) was then performed. The procedure could be completed without circulatory collapse with an additional 0.01 mg bolus of noradrenaline during pre-dilatation balloon aortic valvuloplasty and deployment under rapid pacing. The surgery was then completed, although vasopressin was temporarily required during the right coronary artery and left circumflex artery bypass. Remimazolam was switched to propofol at 2 mg/kg/h and dexmedetomidine at 0.4 μg/kg/h and the patient was moved to the ICU under sedation with intubation. Total operative time was 210 min and anesthesia time was 281 min. He was extubated the day after surgery and discharged from the ICU on the second postoperative day. He was discharged without complications on postoperative day 34.

### Case 2

The patient was a 92-year-old woman (height, 143 cm; weight, 47.1 kg) with dyspnea on exertion. Echocardiography revealed severe AS with an aortic valve peak velocity of 4.2 m/s, calculated aortic valve area of 0.44 cm^2^, and circumferential left ventricular wall thickening. Left ventricular ejection fraction was 69.4%. Coronary angiography revealed a three-vessel coronary artery lesion. Percutaneous coronary intervention to stenosis at the ostia of the right coronary artery was deemed difficult. Other complications included severe bilateral lower artery stenosis, severe stenosis of the right carotid artery, chronic atrial fibrillation, old cerebral infarction, hypertension, and dyslipidemia.

**Fig. 2:**
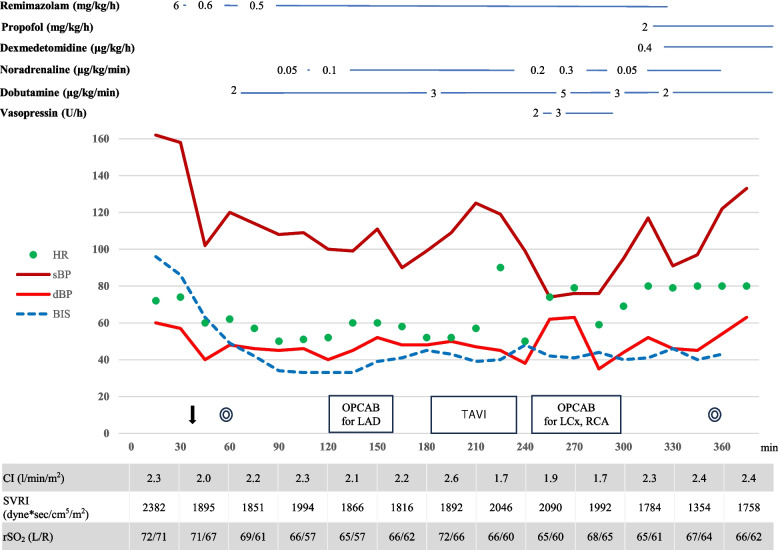
Anesthesia record for case 2. The down arrow represents intubation. Double circles represent the start/end of surgery. HR: heart rate; sBP: systolic blood pressure; dBP: diastolic blood pressure; BIS: bispectral index; CI: cardiac index; SVRI: systemic vascular resistance index; rSO_2_: regional cerebral oxygenation measured by near-infrared spectroscopy; CABG: coronary artery bypass grafting; TAVI: transcatheter aortic valve implantation; LAD:left anterior descending; LCx: left circumflex artery; RCA:right coronary artery

The surgical and anesthesia strategy was the same as that for case 1. General anesthesia was induced with a total of 300 µg of fentanyl in divided doses and remimazolam at 6 mg/kg/h without any vasopressor. After the patient lost consciousness, 1 mg/kg rocuronium was administered. No significant circulatory changes were observed during tracheal intubation. Anesthesia was maintained with a total of 1000 μg fentanyl in divided doses and remimazolam adjusted to 0.3–0.4 mg/kg/h in order to achieve BIS values between 40 and 60. and remimazolam adjusted to 0.5–0.6 mg/kg/h in order to achieve BIS values between 40 and 60. Hemodynamic change during surgery is shown in Fig. 2. During coronary artery bypass to the LAD, hemodynamics was stable with CI 2.1 L/min/m^2^ and SVRI around 1800 dyne sec/cm^5^ with the administration of 0.1 μg/kg/min noradrenaline and 2 μg/kg/min dobutamine. TAVI using a self-expanding supra-annular bioprosthetic valve (Evolut pro; Medtronic Inc., Minneapolis, MN, USA) was then performed. The procedure could be completed without circulatory collapse with an additional 0.01 mg bolus of noradrenaline during deployment under control pacing and three rapid pacing. The surgery was then completed although high-dose catecholamines including vasopressin were temporarily required during the right coronary artery and the left circumflex artery bypass. Remimazolam was switched to propofol at 2 mg/kg/h and dexmedetomidine at 0.4 μg/kg/h and the patient was moved to the ICU under sedation with intubation. The total operative time was 283 min, and the anesthesia time was 360 min. She was extubated the day after surgery and discharged from the ICU on the second postoperative day. She was discharged without complications on postoperative day 18.

## Discussion

Remimazolam provides favorable circulatory stability and is safely used in cardiac surgery [8–10]. Its validity in the induction and maintenance of anesthesia in patients with AS is accumulating [11, 12]. Nakanishi et al. reported safe induction with remimazolam in surgical aortic valve replacement or TAVI in elderly patients [13]. Miyoshi et al. reported that remimazolam was associated with less vasopressor use in the induction and maintenance of TAVI [14]. In hybrid surgery of TAVI and OPCAB, Hashimoto et al. reported anesthetic management with propofol target-controlled infusion for an 86-year-old man [15]. However, anesthetic management with remimazolam in hybrid surgery, especially in very elderly patients, has not been reported.

If TAVI is performed in AS patients with concomitant CAD, there is concern about the risk of myocardial ischemia induced during rapid pacing. Therefore, it was often reported that coronary artery blood flow is first secured by off-pump bypass to the LAD, followed by TAVI to remove cardiac output restriction, and then complete revascularization is performed if necessary [16]. In this procedure, AS and CAD coexist from the induction of anesthesia to the completion of TAVI, and it is, therefore, important to maintain adequate cardiac output and systemic vascular resistance to avoid an imbalance in oxygen supply and demand to the myocardium. Anesthesia was successfully induced in the first case with remimazolam using only a small dose of continuous noradrenaline, and in the second case, any vasopressor was not used during anesthesia induction. In both patients, appropriate circulatory dynamics were maintained until the completion of TAVI, although catecholamine support was required. It is not clear to what extent remimazolam contributed to the maintenance of circulation since the patients were very high-risk patients in which catecholamines were mandatory. Remimazolam maintains a heart rate better than propofol [17]. This property would contribute to the maintenance of cardiac output in AS patients with limited stroke volume. It was reported that overall vasopressor use is less with remimazolam than with propofol or sevoflurane in TAVI [14]. This less vasopressor use may indicate that adequate blood pressure can be achieved without increasing cardiac workload. We believe that the maintenance of hemodynamic stability with the use of remimazolam played an important role in the prevention of hypotension in hybrid surgery.

Remimazolam is generally administered at 12 mg/kg/h during induction, but we administered it at 6 mg/kg/h and achieved rapid onset of sleep without significant changes in hemodynamics. The pharmacokinetics of single doses of remimazolam are not affected by aging [18]. However, in the elderly, individual differences in pharmacodynamic effects are large depending on their comorbidities, and induction at lower doses may therefore be recommended. In elderly high-risk patients, it was reported that there was no difference in the incidence of hypotension with remimazolam at 12 mg/kg/h and 6 mg/kg/h, but induction at 6 mg/kg/h was shown to potentially reduce hypotension only in patients 65 years of age or older [7]. Nakayama et al. reported induction at extremely low doses of 1.0–1.2 mg/kg/h in very elderly patients [19]. Future studies are needed to determine the appropriate induction dose for very elderly patients.

The doses of remimazolam needed to maintain BIS values at 40–60 were 0.3–0.5 mg/kg/h in the patients of this report. The doses were slightly less than those in a previous report: 0.6 mg/kg/h remimazolam for elderly high-risk patients [7]. In the Japanese Phase II study, the doses that achieved BIS values less than 53 were 0.7 mg/kg/h for elderly patients and 1.0 mg/kg/h for non-elderly patients [18]. Since the pharmacodynamics of remimazolam are expected to be enhanced in elderly patients even during maintenance, the continuous dose should be adjusted for each patient. Remimazolam has a shorter context-sensitive half-time than that of midazolam, another benzodiazepine that has a half-life of about 7 min after infusion for about 4 h [20]. This characteristic allowed for rapid adjustment of the appropriate dosage for our very elderly patients.

In conclusion, remimazolam is a safe option for induction and maintenance of general anesthesia in very elderly patients undergoing hybrid surgery. However, the efficacy of remimazolam in high-risk elderly patients undergoing cardiac surgery is still insufficient, and further evidence needs to be accumulated.

## Data Availability

Data sharing is not applicable to this article as no datasets were generated or analyzed during the current study.

## Abbreviations

TAVI: Transcatheter aortic valve implantation
OPCABG: Off-pump coronary artery bypass grafting
CAD: Coronary artery disease
AS: Aortic valve stenosis
LAD: Left anterior descending artery
ABP: Arterial blood pressure
BIS: Bispectral index
SAVR: Surgical aortic valve replacement
